# The Canton Hospital at Geneva

**Published:** 1895-09-07

**Authors:** 


### The Institutional Workshop.

### WITHIN THE HOSPITALS.

### THE CANTON HOSPITAL AT GENEVA.

(By a Correspondent.)

On the south-west borders of Geneva is the hospital for the canton. It is plain in appearance, but well built and contains 350 beds. Pleasantly situated, from the windows at the back are seen the fissured slopes of Mont Salève, where the picturesque contrast of grey shades boldly breaking the general brownish hue of the mountain frequently caused us to pause and admire on the bright afternoon of our visit. Having glanced at the operating-room, we entered a glass-covered corridor running along the back of the building, and passed several convalescent patients sitting about on our way. We mounted to the next floor on which the medical wards are situated. Here a feature of some interest presented itself. We saw a similar glass-covered corridor put to some use. The day was May 1st, and the spring sun shone in with almost Algerian brightness. The sunshine, however, was not to be allowed "to waste its sweetness," for ranged along the corridor were the beds of several patients, who were too ill to leave them. But if the staff of the hospital in Geneva believe in sunshine, they believe still more in fresh air. Dr. Audeoud, who was kindly showing me round, directed my attention to five buildings in the grounds. They were wooden tile-roofed erections, with removable sailcloth sides, and floors raised about three feet from the ground. Into these dry and airy wood-and-sailcloth buildings in another fortnight all the surgical beds would be moved, and would remain there until September 15th. The surgical division of the hospital is thus for the half-year out in the open air.

The provisions made for the use of air and sunlight are striking and novel to a visitor from England, but in other details the hospital perhaps does not compare very favourably with those at home. The wards have not the cleanly cheerfulness of most English hospital wards, and the use of high spring mattresses, the springs of which are visible, gives an impression of carelessness as to appearance. On entering a surgical ward one's ideas of hospital discipline may receive something of a shock when one sees here and there patients confined to bed enjoying their pipes or cigars. A glance at the hospital rules, however, shows that the practice is not allowed unless permission is given by the physician or surgeon, and the would-be smoker must also obtain the consent of his fellow-patients.

In a medical ward a surprise of a different kind greeted us. A gentleman dressed with white apron, like an English barber, was busy at a bedside. We walked up to him and introductions were given. This gentleman proved to be a physician spending the afternoon taking notes of his cases, and this energy was displayed in a hospital with which a school possessing 350 students is connected. The difference in amount of time given by English and Continental physicians to their wards evidently strikes a foreigner. A doctor seen later in the day commented upon this point. He had spent several months in London, and said that he was surprised how seldom the physicians go round their wards. It was not love of their work, he said, but the acquisition of money that was the ruling passion. It was explained to him that the staffs of English hospitals had to make a living, while the Continental physicians are paid sufficiently well to be almost independent of practice; and this not being the case in England, English medicine and medical education consequently suffer.

The nursing staff at the Geneva Hospital does not leave an altogether favourable impression. Pleasant, good-natured faces the nurses have, but they are mostly short and stout, and do not present an appearance of either activity or aptitude. The black dress and Sister Dora-like cap worn by them I understood to be the uniform of a Protestant sisterhood, the centre establishment of which is at Berne. That centre apparently controls the nursing arrangements of all the Swiss Protestant hospitals—a somewhat unfortunate system, since nurses are changed from place to place as the lady superintendent thinks desirable; and no sooner may a nurse become thoroughly conversant with the ways and methods of some member of the medical staff than she may be called away. The nurses are generally drawn from the uneducated classes, a fact that did not please the doctor who had remarked upon the lack of interest shown by English physicians in their wards. Commenting on English nurses, he said that there could be no doubt that they were the best in the world. Their intelligence pleased him, and, curiously enough, his interest was first aroused, not by seeing them at work, but during a period of recreation. Apparently to his surprise, he saw two nurses in the National Gallery admiring pictures by the old masters, thus giving an evidence of education and taste.

On visiting the out-patient department, another Continental feature presented itself—male and female medical students working side by side, and both equally energetic in endeavours to add to their store of knowledge.

In the same out-patient room was a simple apparatus not noticed by the writer in England, although he cannot say that in some hospitals it is not used. That apparatus was a weighing-machine for babies under the age of one year, a padded bassinet-like arrangement being provided for their reception. Above the weighing-machine was a table showing the average increase in weight, week by week, from the time of birth.

Dr. Audeoud afterwards kindly showed me the excellent new pathological buildings behind the hospital for the use of the medical school, and thus ended an interesting visit to this Swiss hospital.

